# Breeding phenology in *Rana temporaria*. Local variation is due to pond temperature and population size

**DOI:** 10.1002/ece3.2356

**Published:** 2016-08-03

**Authors:** Jon Loman

**Affiliations:** ^1^ Deptartment of BiologyLund UniversityLundSweden

**Keywords:** Breeding migration, frogs, spawning

## Abstract

Frog breeding phenology in temperate zones is usually compared to progress of spring temperatures at a regional scale. However, local populations may differ substantially in phenology. To understand this, local climate and other aspects must be studied. In this study, breeding phenology of the common frog, *Rana temporaria*, in a set of ponds in southern Sweden is analyzed. There was within year a variation of up to 3 weeks in start of breeding among local populations. Water temperature was measured in the ponds, and breeding tended to be earlier in warmer ponds (surprise!). Breeding was also earlier in ponds with a large breeding congregation. Alternative reasons for these patterns are suggested and discussed. There was a large residual variation. The common frog has a wide range of acceptable wintering sites, and I hypothesize that the particular choice by a local population may explain part of this residual variation.

## Introduction

In areas with seasonally predictable weather, frogs usually breed more or less seasonally, with different patterns for different species in any one area (Richter‐Boix et al. 2006; Saenz et al. 2006; Walpole et al. 2012). Breeding seems to be triggered by temperature (Navas and Bevier 2001; Oseen and Wasserzug 2002; Arnfield et al. 2012), rainfall (Banks and Beebee 1986; Byrne 2002), and/or even moon phase (Byrne 2002; Grant et al. 2009; Arnfield et al. 2012). Date has also been suggested to influence, usually constraining the season when other factors can effect (Heusser and Ott 1968; Beattie 1985; Reading 1998). In temperate climates, some frogs species' breed early in spring and usually explosively. The short and distinct breeding season makes the registration of breeding data a favorite phenology study subject, and there are a large number of multiyear compilations of the breeding migration (Gittins et al. 1980; Reading 1998; Miwa 2007; Todd et al. 2011), start of calling (Strömberg 1988; Elmberg 1990; Blaustein et al. 2001; Hartel 2008; Lappalainen et al. 2008; Scott et al. 2008), or time for spawning (Elmberg 1990; Beebee 1995; Gollmann et al. 1999; Blaustein et al. 2001; Tryjanovski et al. 2003; Hartel 2008; Scott et al. 2008; Carroll et al. 2009; Neveu 2009; Loman 2014). The choice of variable seems (sensibly) to mainly depend on what is more practical with different species. The record data series is for *Rana temporaria* in Finland 1846–1986 (Terhivuo 1988).

In *Bufo bufo, R. temporaria*,* Rana dalmatina,* many studies have found an effect of various temperature variables on the yearly variation in breeding start (Reading 1998; Gollmann et al. 1999; Sparks et al. 2007; Hartel 2008; Neveu 2009; Loman 2014). The present focus on effects of climate change has prompted the analysis of trends in breeding of these species. A trend to earlier breeding has been found in several studies (Terhivuo 1988; Gibbs and Breisch 2001; Lappalainen et al. 2008; Scott et al. 2008; Neveu 2009; Loman 2014), but in some cases there was no trend (Blaustein et al. 2001; Gibbs and Breisch 2001; Reading 2003; Hartel 2008; Loman 2014) or even a trend to later breeding (Arnfield et al. 2012). These species (all found on the Northern Hemisphere) also tend to breed earlier in more southern locations (Terhivuo 1988; Carroll et al. 2009).

Few studies have, however, studied breeding phenology in a set of localities in one area. Heusser and Ott (1968) observed that the timing of the *B. bufo* breeding migration differed between two neighboring populations. Compilations by Savage (1961) showed large variation in breeding start among populations of *R. temporaria* in the same area. In a previous study on the phenology of *R*. *temporaria* and *R. arvalis* (Loman 2014), I found for both species a pond effect on the time for spawning and (but only for *R. arvalis*) also a trend for earlier breeding. In the present study, I approach the question: “Why is breeding consistently earlier in some ponds than in others?”

## Methods

### Monitoring frog spawn

**Figure 1:**
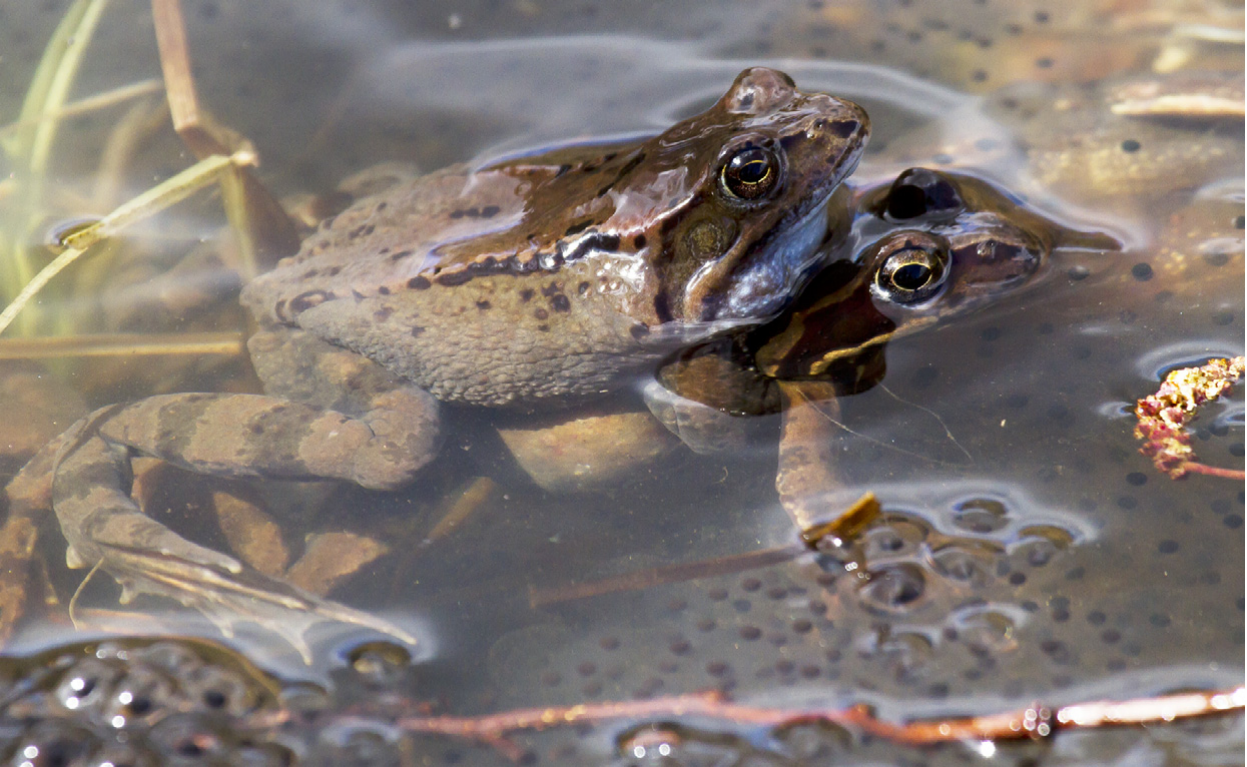
Pair of *Rana temporaria* among fresh spawn.

**Figure 2:**
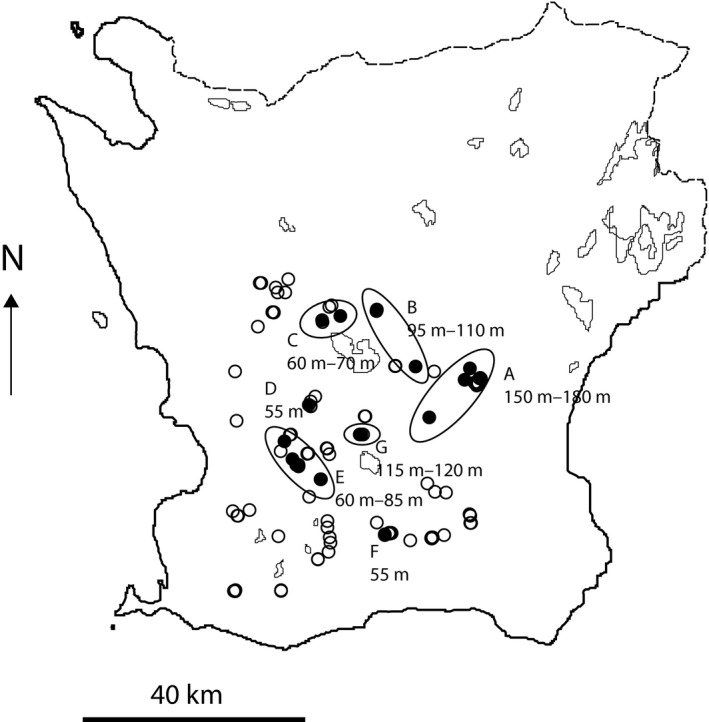
Map of Skåne in south Sweden with study ponds. Filled ponds are those 19 where spring water temperature was monitored with loggers in 2011–2015. Three pair of ponds overlap closely and are difficult to separate. The altitude of the ponds is from 20 m.a.s.l (55 m. for those with temperature monitors) to 180 m. Ovals group ponds close and at similar altitude accounted for in Figure 6. The respective altitude span is shown.

Since 1990 I have monitored breeding time for the common frog *R. temporaria* L. (Fig. 1) in a varying number of ponds in southern Sweden (the province Skåne). The furthest distance between ponds has been 60 km (Fig. 2). Over the years, the number of ponds has varied between 29 and 84. For details until 2010, see Loman (2014). From 2011 until 2015, 29 of those 30 monitored in 2010 have remained in the monitoring scheme.

Within ponds, “breeding sites” were identified. A breeding site consists of all spawn with spawn clumps separated by no more than 1 m. Usually, all clumps at a site were actually in physical contact. The number of spawn clumps was counted visually. However, for very large breeding sites, the area covered with spawn was measured and used to estimate the number of spawn clumps (Loman and Andersson 2007).

For each breeding site, the first date of breeding was recorded. This date usually was a good approximation of the time of breeding for all frogs because at any one site, most frogs bred in the first 2 days (pers. obs; Loman and Håkansson 2004). Ponds were visited about every 5 days during the breeding period. Time for the earliest spawn at a site could therefore be extrapolated from the condition of the spawn at the time of visits. This can usually be performed with a certainty of one or at most 2 days. Another reason for the frequent visits was the need to distinguish spawn of *R. temporaria* from that of *R. arvalis,* a species that often breeds in the same ponds or even share breeding sites. After more than 5 days, this tends to be difficult.

For each pond, breeding time was computed as the average breeding time for all sites in the pond, weighted by the number of female frogs breeding (equal to number of spawn clumps) at each site. Thus, the measure approximated the actual average breeding time for all frogs at each pond. For ponds with several breeding sites, this measure should be more stable than overall date (first breeding of any frog in the pond), which is usually what is recorded in frog phenology studies. Most ponds had only one or two breeding sites.

### Temperature measurements in ponds

In 19 of those 30 ponds (Fig. 2) that were monitored 2011–2015, temperature was measured using loggers (MEgic Disk, Mühlhouse Electronics). The loggers recorded temperature every 2 h. They were placed in a small vacuumed plastic bag and fixed with a rubber band under a floating piece of wooden board (25 × 12 cm, 2 cm thick). There was a 4‐cm hole in the board. A bamboo stick through the hole and stuck into the pond bottom prevented the board and logger from moving horizontally, but it stayed at the water surface. The recorded temperature thus represents water temperature just below the surface, in shadow. Breeding sites tend to remain at the same place from 1 year to the next but changes are frequent. I thus placed the loggers at likely places, about one meter from the shore and above a depth of about 10–20 cm. Occasionally, ponds dried up faster than anticipated; the loggers and bamboo sticks were then moved out, not to become stranded. In each pond, two or occasionally one, or three loggers (Table 1) were placed each year. The target was two but there were failures, and when I had a surplus, I used all available. The loggers recorded temperature every second hour. The average of all values during the period all loggers were out (Table 1) was used for each logger. Each pond and year is represented by the average value for the pond's (remaining) loggers (1–3). Logger ponds represented different regions and altitudes of the study area (Fig. 2). Variation in pond temperature (as recorded by loggers) was related to region and altitude, but this was far from clear cut. Some relatively warm ponds were found in the high‐altitude areas A, B, and G, and a relatively cold pond was found in the more low‐lying area E (Fig. 2).

**Table 1:** Number and exposure of temperature loggers in the years 2011–2015

	2011	2012	2013	2014	2015
Loggers out	25/3	13/3	5/4	7/3	28/2
Ice gone	1/4	19/3	16/4	No ice	No ice
First breeding	1/4	23/3	15/4	19/3	28/3
Latest breeding	16/4	12/4	23/4	7/4	25/4
Loggers in	14/4	9/4	24/4	7/4	10/4
Three loggers (no. of ponds)	0	0	6	3	1
Two loggers (no. of ponds)	18	13	12	9	12
One logger (no. of ponds)	1	6	1	7	5
Missing (no. of ponds)	0	0	0	0	1

“Loggers out” is the date when the last logger was in place and “Loggers in” when the first logger of the year was retrieved. “Ice gone” refers to the date when ice had disappeared from the last pond in the year. “First breeding” may take place earlier, in a warmer pond.

Because the loggers' battery life was limited and estimated to about 6–7 weeks, I tried to time the field period of the loggers to the spring's progress of the respective year and the anticipated start of frog breeding. In 2011–2014, this resulted in temperature recordings starting 7–12 days before first breeding in any one pond. February 2015 was unusually warm, and I felt I had to put out the loggers already late February. However, March weather returned to normal and first breeding took place when loggers had been already out for 30 days. Loggers were usually retrieved about the time of breeding start in the latest pond. However, in 2015, they were retrieved earlier not to risk a battery failure. At that time (April 10th), breeding had not yet begun in three ponds.

## Results

In all years, there was a strong correlation between the reading (average daily mean temperature) of the first and second logger (2011: *N* = 18, *r* = 0.91; 2012: *N* = 13, *r* = 0.89; 2013: *N* = 18, *r* = 0.92; 2014: *N* = 12, *r* = 0.86; 2015: *N* = 12, *r* = 0.84; all *P* < 0.001).

**Figure 3:**
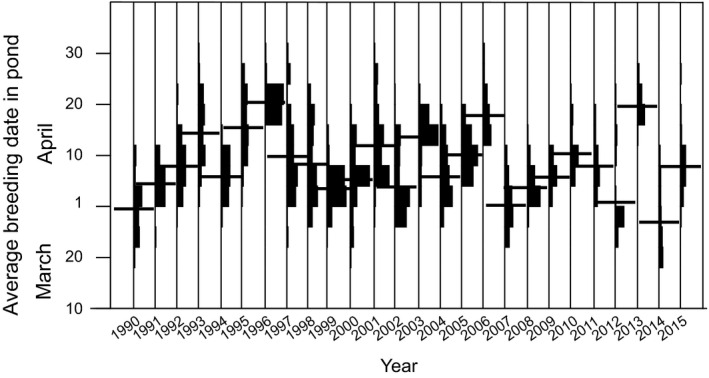
Breeding phenology for all ponds 1990–2015. Vertical histograms show number of ponds in respective year. Bars show average date for year.

**Figure 4:**
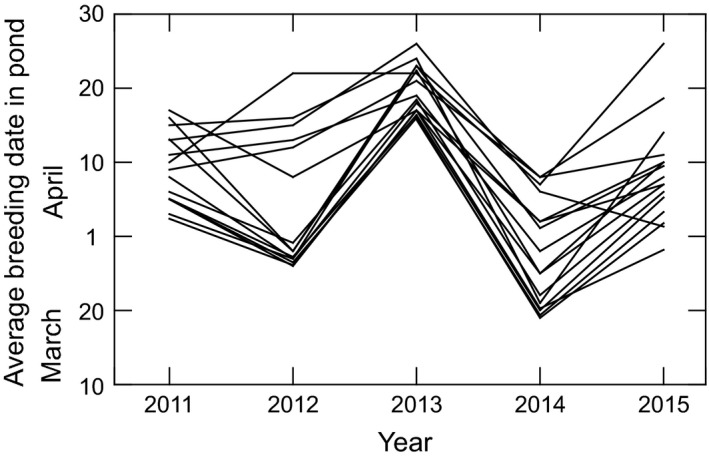
Breeding phenology 2011–2015 for 19 ponds monitored with temperature loggers. Dates for each pond connected by lines.

There were strong pond and year effects on breeding time (2‐factor ANOVA; year: df = 25, *F* = 89.0, *P* < 0.0012; pond: df = 149, *F* = 6.73, *P* < 0.001) (Fig. 3). This result is based on all monitored ponds since 1990. It is also obvious for a subset; those ponds where loggers were used in 2011–2015 (Fig. 4).

There were also strong pond and year effects on pond temperature, as recorded by loggers (2‐factor ANOVA; year: df = 4, *F* = 26.4, *P* < 0.001; pond: df = 18, *F* = 10.2, *P* < 0.001).

**Figure 5:**
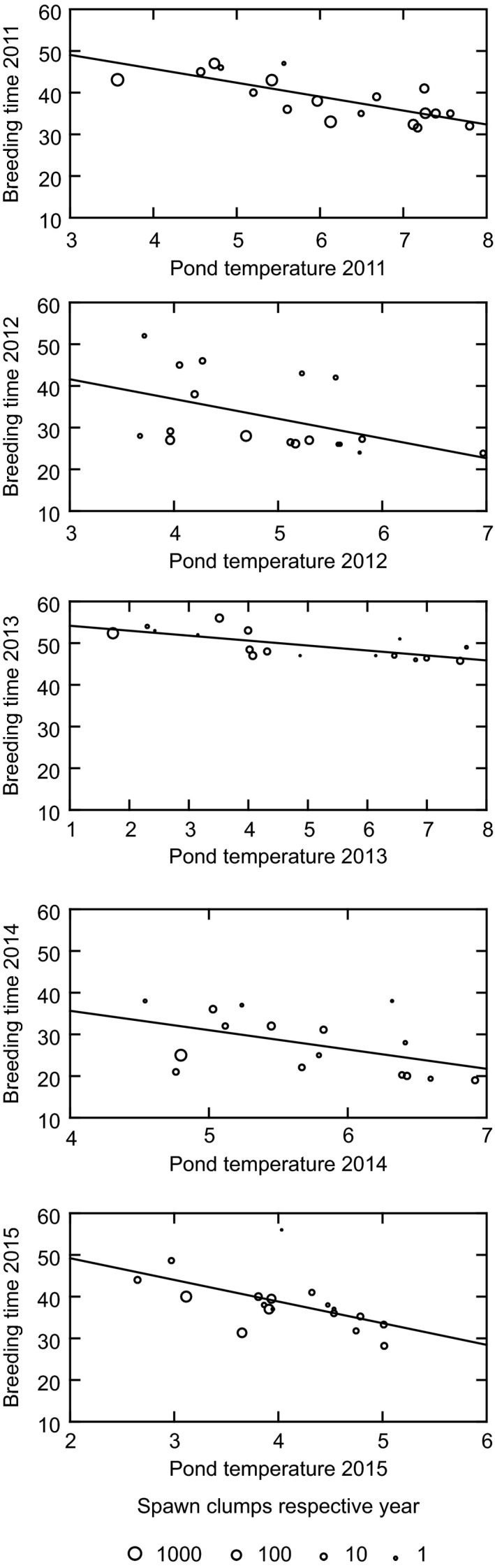
Breeding time in relation to pond temperature and size of breeding congregation. Breeding time is given as days after March 1. Temperature index is mean daily temperature from loggers during the period of monitoring (different for different years). Population *s*ize is estimated as number of spawn clumps in the pond.

There were effects of pond temperature (average logger value for 2011–2015) and number of breeding frogs (average spawn clump number 2011–2015) on average breeding time in 2011–2015 (mult. regression; temperature: *t* = 4.31, *P* = 0.001; number: *t* = 3.00, *P* = 0.008). Stated differently, some ponds are consistently early and others late. Early ponds are relatively warm and/or attract large number of breeding frogs (Fig. 5).

**Figure 6:**
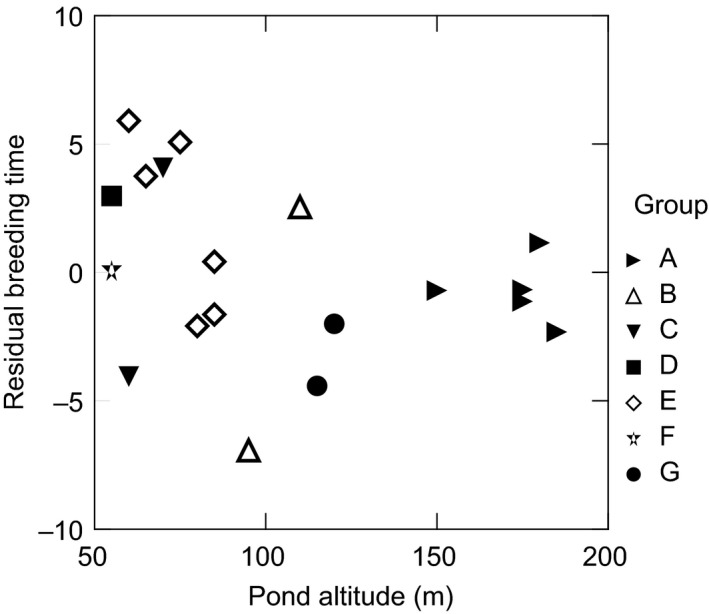
Effect of pond group (Fig. 2) and pond altitude on residual breeding date (when effects of pond temperature and breeding congregation size have been accounted for). Each data point is one pond.

Accounting for temperature and number of breeding frogs, there was still a span of pond phenology, with residual days for an early pond −7 days to a late pond with a residual +6 days (Fig. 6).

## Discussion

### What do the logger temperatures represent?

The loggers were in the field during different dates in the different years. Also, there was no fixed, consistent relation between the start of breeding and the logged dates. Because the temporal pattern of breeding differed among years (Fig. 3) and it is not known what actually triggers breeding, it had been difficult to define such a relation. During the period immediately before and during breeding, the average temperature, as measured by the logger, represents the pond temperature as potentially experienced by breeding frogs. It should correctly rate ponds from warm to cool. However, the temperatures cannot be used to compare the spring temperatures of the 5 years. Such an analysis based on air temperatures, for *R. temporaria* in the same set of ponds, shows (not surprisingly) that these frogs breed earlier in years when March is relatively warm (Loman 2014).

It was not possible to beforehand decide exactly where the frogs would breed. The loggers were thus placed at places that in my experience represent typical breeding sites of *R. temporaria* in the respective ponds. In many cases, it actually turned out they bred in the immediate vicinity. Temperature variation within ponds was much less than that among. Thus, both single logger readings and the average of two or three, when available, should be a useful representation of temperature in the respective pond at sites where frogs typically breed.

### Pond temperature effects

A temperature effect on breeding start is not surprising and has also been found in numerous studies (many examples are found in While and Uller (2014)). What sets the present study off from most previous is that I, like Elmberg and Lundberg (1991) and Scheckenhofer et al. (2011), have measured water temperature, rather than air temperature. Also, I have measured temperature in the individual ponds rather than a regional average. Air temperature differs little regionally among ponds, and the average March and April temperature differs with less than a degree over my study area, compared to a mean difference of 3.1°C between my warmest and coldest pond. There is an obvious scope for a causal effect on time for spawning. Breeding early should to some degree result in early hatching of eggs and metamorphosis of tadpoles (Loman 2009). In turn, this could give the offspring a head start (Cabrera‐Guzman et al. 2013). However, breeding too early increases the risk for cold periods with ice cover that could damage laid spawn (Frisbie et al. 2000; Loman 2009) and in extreme cases also tadpoles (Muir et al. 2014). Actual pond temperature could be used by the frogs as a clue to an optimum balance. Alternatively, a more direct effect of temperature comes from the temperature dependence of frog movement (Whitehead et al. 1989); at low temperatures, risk of falling victim to predation increases, especially during a conspicuous activity such as breeding. Finally, the temperature effect could be physiological in more direct sense if sperm and/or eggs need a certain amount of warmth to mature.

### Effect from number of breeding frogs

Breeding tended to be earlier if there were many spawn clumps in the pond. The effect size found means that with 5 breeding males, breeding can be expected to be 7.2 days later than with 500 so this is potentially an important aspect. The number of spawn clumps represents the number of breeding females (Loman and Andersson 2007), and we can expect a strong correlation between this and the number of breeding males. Assuming that individual target days for breeding are normally distributed, it is clear that with a large number of males, there will be more early breeders. Calling at a breeding site can start several days before the laying of first spawn (pers. obs.) although this is not universal (Hartel et al. 2007). Therefore, another possibility is that (potentially) late breeders are triggered by early ones and the early ones thus set the standard for the whole breeding congregation. This would thus by itself result in earlier breeding in large congregations. A slightly different mechanism could be that males somehow sense the size of the congregation (especially if calling begins before spawning) and being in a large one are more likely to start calling early. This mechanism would also affect early breeding frogs, bringing breeding further forward. Reasons for this could be that competition is likely to be important, favoring an early start even if calling could attract predators to the calling frog. Another reason could be that in a larger congregation temperature in the communal spawn, mass is higher (Loman and Håkansson 2004) and the risk associated with cold weather and freezing water early in the season is less. Timing could also be triggered by the females who supposedly have the ultimate control of spawning. If many frogs are calling, this may trigger females to breed earlier. Actually, it is common to hear calling frogs at a site several days before the first spawn is laid (pers. obs.). With lack of more knowledge on actual frog behavior at the breeding sites, it is not possible to decide among these suggestions.

### Other effects

The large residual effects (Fig. 6) suggest that more factors than temperature and size of breeding congregation are important for timing the spawning. However, from Figure 6, one cannot conclude that region or altitude is likely to be one. The latter is in accord with findings by Muir et al. (2014). I have no further data to support any hypothesis but would like to suggest one. Spawning is preceded by the breeding migration. *R. temporaria* is broad in its choice of wintering sites, and it is known to spend the winter in ponds, even under ice (Ashby 1969; Koskela and Pasanen 1974; Pasanen et al. 1993; Pasanen and Sorjonen 1994; Boutilier et al. 1999; Ludwig et al. 2013; pers. obs.), in soil (Ashby 1969; Pasanen and Sorjonen 1994) as well as in running water (Thomas Madsen pers. comm.). It is not known where the frogs in the study populations winter but one could suppose that for those wintering close to the breeding pond, or even in it, spawning is closely coupled to the pond temperature while frogs that have to migrate may well be delayed, depending on the occurrence of suitable migration weather. Migration takes predominantly place during night and is thus more dependent on night temperature (and possibly moisture) than average diel air temperature that affects pond temperature. This hypothesis is discussed by Savage (1961) who, however, refutes it on ground that were it true, there would be a clear dichotomy between early (frogs wintering in the breeding pond) and later (frogs migrating to the breeding pond). He did not find this (nor did I), but assuming a range of migration distances and conditions and further factors, I suggest there is still scope for a substantial effect from the respective populations' wintering conditions.

### The importance of local phenology

The findings of this study stress the fact that phenology variation has an important local component. This has received surprisingly little attention but some examples include hibernation penology of a ground squirrel *(Urocitellus parryii)* (Sheriff et al. 2011), an alpine plant *(Anthyllis vulneraria)* (Kesselring et al. 2015), and breeding birds in urban and rural habitat (Möller 2015). In contrast, breeding swallows *(Hirundo rustica)* did not respond locally to variation in weather (Grimm et al. 2015).

I draw two conclusions from local variation in phenology. Firstly, studies of local variation can provide clues to various aspects of the species' biology. In the case of *R. temporaria,* it draws the attention to the possible importance of hibernation sites and migration for phenology. For urban birds, it is suggested that comparatively abundant food (bird feeders!) during winter contributes to an early start of breeding. Second, in phenology studies, on must be aware that a single site may be a poor representative of regional phenology, generating noice in interregional comparisons. However, for studies of phenology trends over time, one *single* site may give reasonable results. This is so if relative phenology among local sites is stable over time, some sites consistently early, others late, as in the present stud*y*.

## Conflict of Interest

None declared.
